# Concordant and Discordant Interrelationships of the GERD Triad of Symptoms, Endoscopy Findings, and Histopathological Changes Over Time after One Anastomosis Gastric Bypass

**DOI:** 10.1007/s11695-025-08277-7

**Published:** 2025-11-18

**Authors:** Mohamed Hany, Ahmed Zidan, Kareem El-Ansari, Walid El Ansari

**Affiliations:** 1 Department of Surgery, Medical Research Institute, Alexandria University, Alexandria, Egypt; 2 Alexandria University, Madina Women’s Hospital, Alexandria, Egypt; 3 Faculty of Medicine, St. George’s University, St. George’s, Grenada; 4 College of Medicine, Ajman University, Ajman, United Arab Emirates

**Keywords:** Reflux, OAGB, Endoscopy, Evolution, Biopsy, GERD, Symptoms, Histopathology

## Abstract

**Background:**

Reflux-related symptoms, upper endoscopy (UE), and histopathology findings comprise a triad of changes of gastro-esophageal reflux disease (GERD) following one anastomosis gastric bypass (OAGB). However, the evolution and interplay between these changes across time have not been sufficiently evaluated.

**Methods:**

This study is a retrospective analysis of the GERD triad in 150 patients using GerdQ questionnaire, UE, and histopathology at year 1 and year 3 after OAGB. Evolution of the GERD triad was explored, covering five areas: evolution of changes in the GERD triad over time; relationships and correlations between changes in the GERD triad components in the form of concordance or discordance among these components; subsets of patient demographics or time intervals that necessitate heightened awareness for post-OAGB reflux-related abnormalities; characteristics defining concordant compared to discordant cases; and potential predictors that increase the likelihood of discordance.

**Setting:**

University hospital, Alexandria, Egypt.

**Results:**

Mean age was 34.7 years, with 75.3% females. At year 1, 25.7% were symptomatic, yet 62.5% and 65.3% of patients had UE and histopathology abnormalities respectively. Hence, 36.8% of patients with abnormal UE and 39.6% with abnormal histopathology were asymptomatic. At year 3, 55.6% were symptomatic, yet 75.8% and 78.2% had UE and histopathological abnormalities respectively. Discordant cases comprised 39.6% and 22.6% of patients at years 1 and 3 respectively. There were no significant differences in patient characteristics between concordant and discordant cases at years 1 and 3. Although symptoms were significantly one-third lower at year 1 compared to year 3, the likelihood of discordance was significantly higher at year 1 (OR = 2.81, 95% CI = 1.64–4.80, p < 0.001) and in patients with elevated hemoglobin (OR = 1.37, 95% CI = 1.10–1.71, p = 0.005).

**Conclusion:**

There was variable evolution of the GERD triad. Concordant cases posed no significant clinical threats, while discordant cases require a high index of suspicion. At year 1, there were more asymptomatic patients with more discordance, while the higher likelihood of symptoms at year 3 calls for extended follow up beyond year 3.

**Supplementary Information:**

The online version contains supplementary material available at 10.1007/s11695-025-08277-7.

## Introduction

Obesity remains a global health challenge, and metabolic and bariatric surgery (MBS) is highly effective in managing obesity and related medical conditions [1]. Recently, One-Anastomosis Gastric Bypass (OAGB) has gained traction, primarily due to its favorable effectiveness and safety profiles [2–4]. OAGB has been endorsed by the American Society for Metabolic and Bariatric Surgery (ASMBS) and The International Federation for the Surgery and Other Therapies for Obesity (IFSO), and has shown a surge in popularity according to the IFSO global registries [5–7].

There is an ongoing discourse surrounding post-OAGB gastroesophageal reflux disease (GERD). Aspects such as the correlation between reflux symptoms and corresponding macro- or microscopic abnormalities remain contentious among OAGB patients. Evidence suggests that symptoms alone may not reliably diagnose GERD after OAGB; therefore, routine upper endoscopy (UE) and/or biopsy have been advocated for definitive diagnosis [8, 9].

A prospective mid-term study of OAGB outcomes revealed that even asymptomatic patients exhibited macroscopic and microscopic inflammatory changes in the gastrojejunostomy, gastric pouch, and distal esophagus during UE [10]. Similarly, other studies have reported discordant findings regarding symptoms and histological changes in OAGB patients [11–13]. Despite these observations, no research has comprehensively evaluated the triad of reflux symptoms, UE findings, and histopathological changes longitudinally after OAGB.

The literature highlights several knowledge gaps. Many studies have relied solely on symptoms, observed clinically or through questionnaires, to identify reflux [14–20]. Others utilized a combination of clinical symptoms and UE to detect reflux [9, 21–26]. A common limitation has been the failure to longitudinally assess the interplay between symptomatology, UE findings, and histopathological changes after OAGB, which could yield crucial insights into their evolving relationships over time.Therefore, the current study aimed to address the temporal evolution of this GERD-related triad.

## Materials and Methods

### Study Design and Ethics

This study is a retrospective analysis of prospectively gathered data on OAGB conducted at our institution in Alexandria, Egypt, between December 2020 and February 2022. The research received approval from the institution's Ethics Committee, and all participating patients provided written informed consent.

### Inclusion and Exclusion Criteria

The current series comprised adult patients aged between 18 and 60 of both sexes who underwent primary OAGB during the specified period, with a body mass index (BMI) exceeding 40 kg/m^2^ or above 35 kg/m^2^ with associated comorbidities, aligning with the NIH guidelines [27].

Exclusion criteria included history of previous MBS, major abdominal operations/explorations, preoperative GERD symptoms, ulcers, Barrett's esophagus (BE), smoking, alcohol consumption, UE findings indicating GERD according to the Los Angeles (LA) classification [28], or large hiatal hernia (> 5 cm). All patients underwent H. pylori testing, and eradication was performed if positive [19].

### Study Objectives

These comprised five interconnected issues over the specified follow-up period: 1) *Evolution*: changes in the GERD triad over time; 2) *Relationships*: correlations between the changes in the components of the GERD triad; 3) *Subsets*: specific patient demographics or time intervals that necessitate heightened awareness for post-OAGB reflux-related abnormalities; 4) *Characteristics*: what defines concordant cases (alignment among the GERD triad components) compared to discordant cases (non-alignment among these components); and, 5) *Potential Predictors*: characteristics that increase likelihood of discordance; or time points where probability of discordance is high. This study is, to our knowledge, the first to systematically address these complex issues.

### Data Collection

Data were systematically collected at years 1 and 3 post-OAGB. The year 1 follow-up aimed to capture early post-OAGB reflux instances, concurring with assessments of UE and histopathological changes after OAGB, while the year 3 follow-up aimed to allow sufficient time for potential development of macro/microscopic indicators and to permit the emergence of symptoms. This aligns with the ASMBS recommendations advocating routine endoscopies at specified post-surgical intervals [29], and also with data from studies evaluating post-OAGB and MBS reflux [8, 9, 30].

### Datasets

#### Gastroesophageal Reflux Disease Questionnaire (GerdQ)

GERD symptomatology was evaluated using the validated self-administered instrument, GerdQ, which inquires about six items: heartburn, regurgitation, epigastric discomfort, nausea, sleep disturbances due to heartburn, and utilization of over-the-counter antacids, [31]. Each item is rated with a score of zero to three, reflecting the frequency of symptoms over the preceding week, with a total score 0–18. GerdQ has been widely implemented in the bariatric domain for evaluating GERD [19, 32], and has been corroborated by various studies [11, 19, 31, 33, 34]. Patients scoring ≥ eight points have an 80% likelihood of having GERD, with 65% sensitivity and 71% specificity for diagnosing GERD.

To mitigate inter-rater variability, one bilingual researcher, fluent in Arabic and English, administered the questionnaire to all participants. The researcher underwent thorough training with the study team to deepen comprehension of the GerdQ items, their concepts, and the administration process, ensuring impartiality and avoiding patient coercion during the completion of the questionnaire.

#### Upper Gastrointestinal Endoscopy

UE macroscopically assessed the distal esophagus, gastric pouch, anastomotic site, as well as the presence or absence of bile in the esophagus and gastric pouch. Specimens were biopsied systematically from these three areas for histological examination [29]. Thorough endoscopic examination and biopsies from the three areas were adopted for comprehensive reflux assessment, as bile and acid exposure frequently affect the gastric pouch and the anastomosis, producing symptoms that resemble GERD symptoms. This would be particularly valuable when no endoscopic macroscopic evidence of reflux exists.

#### Biopsy Protocol (Histopathology)

This included four-quadrant mucosal biopsies from the gastrojejunostomy, gastric pouch, and esophagus above the Z-line. If BE was suspected, 8 random biopsies were obtained from the salmon-colored mucosa. When 8 biopsies were unattainable due to short (1–2 cm) segments of suspected BE, at least 4 biopsies per centimeter of circumferential BE, and 1 biopsy per centimeter in the tongue of BE were taken, in line with the ASMBS guidelines [29]. An experienced pathologist conducted the microscopic evaluations of the samples, classifying findings as normal or abnormal at three anatomical levels: distal esophagus (chronic inflammation, BE), gastric pouch (chronic gastritis, erosive gastritis, H. pylori infection), and anastomotic site (chronic inflammation, erosions, ulcers).

#### Surgical Technique

Details of OAGB technique are outlined in Supplementary Box 1.

#### Evolution and Concordance/Discordance of GERD triad Post-OAGB

To assess the evolution as well as the concordance/discordance of GERD triad following OAGB, patients were grouped using various parameters of the GERD triad and whether these parameters aligned with each other at the time points under examination (Box 1).

**Box 1:** Evolution, concordance and discordance of the GERD triad after OAGB

**Evolution:** To assess evolution of the GERD triad, patients were initially grouped using three parameters: symptoms only, diagnostic modality only (UE and biopsy), and a combined analysis of symptoms with diagnostic modalities. The patient distribution at year 1 is presented based on these parameters, with subsequent tracking to explore the evolution of each cohort's status at year 3, including metrics on loss to follow-up, stability of the condition, and changes in status
**Concordance/Discordance:** Patients were further categorized based on their GerdQ score (symptomatic versus asymptomatic), UE findings (normal versus abnormal), and biopsy findings (normal versus abnormal) into two groups: concordant and discordant. Concordance was defined as agreement across symptoms, UE, and histopathology findings; discordance referred to discrepancies/non-agreement among these metrics. Concordant vs discordant status was evaluated at year 1 follow-up, and again at year 3 to explore the alignment status

### Statistical Analysis

Statistical analyses were conducted using R software version 4.2.2 [35]. Descriptive statistics included means and standard deviations (M ± SD) for continuous variables, as well as frequencies and percentages for categorical variables. To explore factors associated with discordance, we performed descriptive comparisons of demographics, comorbidities, and laboratory findings between concordant and discordant cases at postoperative years 1 and 3, utilizing chi-square tests (or Fisher's exact tests) for categorical variables and independent t-tests for continuous variables, highlighting significant differences at the specified time points.

To assess the relationship between patient characteristics, time, and likelihood of discordance, we employed generalized estimating equations (GEE) using the ‘geepack’ package [36] in R Software. This methodology was selected due to its capacity to manage the correlated structure of repeated measures data, providing robust standard errors even in the presence of missing data. The GEE models included time as a categorical variable (year 1 and year 3) along with covariates such as demographic information and laboratory results, using binary logistic links to model the probability of discordance. Variable selection was based on *p*-values < 0.25 in univariate analyses, in line with published recommendations [37] acknowledging that covariates with weak univariate associations may significantly impact the model when combined.

## Results

### Participants

A total of 295 consecutive patients underwent OAGB during the study period. Of these, 145 patients were excluded based on non-compliance with the inclusion criteria, including smokers (n = 26), alcohol consumption (n = 3), overseas patients returning home post-surgery (n = 53), large (> 5 cm) hiatal hernia (n = 6), age > 60 years (n = 23), GERD (LA classification) (n = 17), prior MBS (n = 11), or previous abdominal exploration (n = 6). Consequently, 150 patients were included in the current analysis. By year 1, six patients were lost to follow-up, with an additional 20 lost by year 3.

### Preoperative Characteristics

The cohort was predominantly females (75.3%, n = 113), with a mean age of 34.7 ± 11.4 years (range: 18–60 years), and BMI of 43.8 ± 3.2 kg/m2 (range: 36.0–54.1). All patients were asymptomatic with normal UE findings at the preoperative evaluation. Associated medical problems included dyslipidemia (45.3%), osteoarthritis (39.3%), hypertension (20.6%), sleep apnea (13.3%), diabetes mellitus (8%), and cardiac conditions (4.6%).

### Dynamics of Symptomatology and Diagnostic Findings

Table 1 outlines the incidence of positive GERD symptoms and corresponding diagnostic findings over the study duration. Notably, 36.8% of patients with abnormal UE and 39.6% with abnormal histopathology were asymptomatic, indicating a considerable incidence of 'clinically silent' conditions where detectable macro- and microscopic pathologies existed without overt symptoms. The trend was similar at year 3, where 20.2% and 22.6% of patients had abnormal UE and histopathology findings, respectively, despite maintaining asymptomatic status. Details of positive endoscopic and histopathological findings are provided in Supplementary Table 1.

**Table 1:** GerdQ score, and endoscopic and biopsy findings at two time points after OAGB

Variable	Year 1 (n = 144)	Year 3 (n = 124)	% Change between years 1 and 3
GerdQ score			
Symptomatic (≥ 8)	37(25.7)	69(55.6)	29.9
Asymptomatic (< 8)	107(74.3)	55(44.4)	
Endoscopy (UE)			
Abnormal^*a*^	90(62.5)	94(75.8)	13.3
Normal	54(37.5)	30(24.2)	
Biopsy (Histopathology)			
Abnormal^*b*^	94(65.3)	97(78.2)	12.9
Normal	50(34.7)	27(21.8)	

Cell values represent frequency (%); *GerdQ* gastroesophageal reflux disease symptoms questionnaire; ^*a*^ abnormal macroscopic/endoscopic findings;^*b*^ abnormal microscopic/histopathological findings

### Evolution of Symptoms, Endoscopy, and Biopsy Findings Post-OAGB

Table 2 presents the progression of symptoms, UE findings, and biopsy results over time after OAGB. At year 1, patients were grouped first according to their GerdQ scores into asymptomatic and symptomatic, then further sub-grouped according to the findings of UE and the biopsy histopathology. The changes in patients’ status regarding symptoms, UE findings, and biopsy results were noted at year 3. The table highlights the interrelation of symptoms and diagnostic outcomes and underscores the patients who necessitate closer scrutiny, particularly, the asymptomatic discordant cases.

**Table 2:** Evolution of symptoms, endoscopy findings and histopathology findings of Year1 patients to Year 3

Status at Year 1 (N = 144)		Status at Year 3 (N = 124)	
Asymptomatic patients, n = 107 (74.3%)	n = 50 (34.7%): GerdQ_(-) +_ UE_(-)_ + Biopsy_(-)_	Lost to follow up: n = 5 (3.5%)	
		No change: n = 27 (18.8%)	n = 27 (18.8%): remained GerdQ_(-)_ + UE_(-)_ + Biopsy_(-)_
		Change: n = 18 (12.5%)	n = 6 (4.2%)^a^: became GerdQ_(-)_ + UE_(+)_ + Biopsy_(+)_
			n = 12 (8.3%): became GerdQ_(+)_ + UE_(+)_ + Biopsy_(+)_
	n = 4 (2.8%) ^*a*^: GerdQ_(-)_ + UE_(-)_ + Biopsy_(+)_	Lost to follow up: n = 3 (2.1%)	
		No change: n = 0 (0%)	
		Change: n = 1(0.7%)	n = 1 (0.7%): became GerdQ_(+)_ + UE_(+)_ + Biopsy_(+)_^*^
	n = 53 (36.8%) ^*a*^: GerdQ_(-)_ + UE_(+)_ + Biopsy_(+)_	Lost to follow up: n = 6 (4.2%)	
		No Change: n = 15 (10.4%) ^*a*^	n = 15 (10.4%)^a^: remained GerdQ_(-)_ + UE_(+)_ + Biopsy_(+)_
		Change: n = 32 (22.2%)	n = 3 (2.1%)^a^: became GerdQ_(-)_ + UE_(-)_ + Biopsy_(+)_
			n = 29 (20.1%): became GerdQ_(+)_ + UE_(+)_ + Biopsy_(+)_
Symptomatic patients, n = 37 (25.7%)	n = 37 (25.7%): GerdQ_(+)_ + UE_(+)_ + Biopsy_(+)_	Lost to follow up: n = 6 (4.2%)	
		No change: n = 27 (18.8%)	n = 27 (18.8%): remained GerdQ_(+)_ + UE_(+)_ + Biopsy_(+)_
		Change: n = 4 (2.8%) ^*a*^	n = 4 (2.8%)^a^: became GerdQ_(-)_ + UE_(+)_ + Biopsy_(+)_

All percentages computed as proportions of the year 1 patients (denominator = 144); *GerdQ* gastroesophageal reflux disease symptoms questionnaire; *GerdQ*_*(-)*_ asymptomatic (GerdQ score < 8); *GerdQ*_*(*+*)*_ symptomatic (GerdQ score ≥ 8); *UE* upper endoscopy; *UE*_*(-)*_ negative (normal) endoscopic/macroscopic findings; *UE*_*(*+*)*_ positive (abnormal) endoscopic/macroscopic findings; *Biopsy*_*(-)*_ negative (normal) histopathological/microscopic findings; *Biopsy*_*(*+*)*_ positive (abnormal) histopathological/microscopic findings; ^***a***^ Discordant patients; ^*^ this patient was Biopsy_(+)_ at gastric pouch only at year 1 and year 3, but progressed to Biopsy_(+)_ at distal esophagus and became UE_(+)_ at gastric pouch; bold text indicates variable/s where change in status was detected at year 3

From year 1 to year 3, some patients remained stable in their symptom/UE/biopsy status, while others experienced changes between asymptomatic and symptomatic states, or from normal to abnormal UE or histopathology. Discrepancies between UE and histopathology outcomes were infrequent, amounting to 2.8% at year 1 and 2.1% at year 3 as histopathology-positive but UE-negative. The cumulative discrepancy rate across both time points was low at 2.6%.

### Patients Requiring High Index of Suspicion: Concordant and Discordant Cases

Table 3 demonstrates the concordance and discordance of patients according to their symptoms, UE, and biopsy findings at year 1 and their longitudinal evolution at year 3 to addresses the reliability of symptoms at these two time points and the necessity of UE and biopsy in each context. This section delineates post-OAGB patient groups that warrant heightened vigilance regarding reflux-related abnormalities.

**Table 3:** Changes to concordant and discordant Patient subgroups and their clinical implications

Year 1 Status (n = 144)				Change in Status at Year 3 (n = 124)				Caution points
Patient group	SS	UE	B	Patient group	SS	UE	B	
A) Concordant at year 1, n = 87/144 (60.4%)								
Asymptomatic patients, n = 50 (34.7%)^*a*^: GerdQ_(-)_ + UE_(-)_ + biopsy_(-)_	D	O	C	6 patients (4.8%)^*b*^ remained asymptomatic GerdQ_(-)_ but turned to UE_(+)_ + biopsy_(+)_	ND	M	C	Could be missed at year 3 if either UE or biopsy not done
				12 patients (9.6%) ^*a*^ became symptomatic GerdQ_(+)_ + UE_(+)_ + biopsy_(+)_	CBD	O	C	Should not be missed at year 3
Symptomatic patients, n = 37(25.7%)^*a*^: GerdQ_(+)_ + UE_(+)_ + biopsy_(+)_	D	O	C	4 patients (3.2%)^*a*^ became asymptomatic GerdQ_(-)_ + UE_(+)_ + biopsy_(+)_	ND	M	C	Could be missed at year 3 if UE and/or biopsy not undertaken
				*Total change from Concordant to Discordant at Year-3, n* = *(6* + *4)/124 (8.1%)*				
B) Discordant at year 1, n = 57/144 (39.6%)								
Asymptomatic patients, n = 53 (36.8%) ^*b*^*:* GerdQ_(-)_ + UE_(+)_ + biopsy_(+)_	ND	M	C	15 patients (12.1%)^*b*^ remained asymptomatic GerdQ_(-)_ + UE_(+)_ + biopsy_(+)_	ND	M	C	Can be easily missed at year 1 and year 3, require high index of suspicion
	ND	M	C	3 patients (2.4%)^*b*^ remained asymptomatic GerdQ_(-)_ but UE_(-)_ + biopsy_(+)_	ND	ND	M	Will be missed in year 1 if UE not undertaken, and in year 3 if biopsy not undertaken
Asymptomatic patients, n = 4 (2.8%)^*b*^: GerdQ_(-)_ + UE_(-)_ + biopsy_(+)_	ND	ND	M	1 patient (0.8%)**^b^ became symptomatic GerdQ_(+)_ + UE_(+)_ + biopsy_(+)_	CBD	O	C	Should not be missed at year 3
				*Grand total Discordant cases at year-3, n* = *(15* + *3)/124 (14.5%)*				

*GerdQ* gastroesophageal reflux disease symptoms questionnaire; *GerdQ*_*(-)*_ asymptomatic (GerdQ score < 8); *GerdQ*_*(*+*)*_ symptomatic (GerdQ score ≥ 8); *SS* symptoms *UE* upper endoscopy; *UE*_*(-)*_ negative (normal) endoscopic/macroscopic findings; *UE*_*(*+*)*_ positive (abnormal) endoscopic/macroscopic findings; *Biopsy*_*(-)*_ negative (normal) histopathological/microscopic findings; *Biopsy*_*(*+*)*_ positive (abnormal) histopathological/microscopic findings; ^***a***^ concordant patients; ^***b***^ Discordant patients; *D* Dependable; *ND* Not dependable; *M* Mandatory; *O* optional; *C* confirmatory to identify the type of abnormality; *CBD* Could be dependable; ^**^ this patient was Biopsy_(+)_ at gastric pouch only at year 1 and at year 3, progressed to Biopsy_(+)_ at distal esophagus and became UE_(+)_ at gastric pouch; bold text indicates variable/s where change in status were detected at year 3

As shown in Table 3, six (4.8%) patients who were asymptomatic and disease free (concordant) at year 1, developed UE and histopathological abnormalities at year 3 while being still clinically free/asymptomatic (discordant). Moreover, four (3.2%) patients were symptomatic with UE and biopsy abnormalities (concordant) at year 1, but became asymptomatic at year 3, still with positive UE and histopathological abnormalities (discordant). Furthermore, one (0.8%) patient who was asymptomatic, with negative endoscopy but positive biopsy at year 1, maintained the asymptomatic state at year 3 despite developing positive endoscopic findings in addition to their positive biopsy findings. Hence, patients could develop UE or histopathological abnormalities while keeping their asymptomatic status, highlighting the importance of UE and biopsy at year 3, especially for asymptomatic patients.

On the other hand, developing symptoms at year 3 was an indicator for developing UE or histopathological abnormalities, as seen in 12 (9.6%) patients who developed symptoms and UE and biopsy abnormalities (concordant) at year 3 when previously asymptomatic and disease-free (concordant) at year 1. Discordant findings diminished from 39.6% at year 1 to 22.6% [(6 + 4 + 15 + 3)/124] by year 3.

### Characteristics of Concordant and Discordant Cases at Two Time Points

Table 4 summarizes the characteristics of concordant and discordant cases at year 1 and year 3, focusing on symptoms, demographics, anthropometry, and associated medical conditions. At year 1, all discordant cases (100%) were asymptomatic, in contrast to 57.5% of concordant cases being asymptomatic (p < 0.001). By year 3, discordant cases remained entirely asymptomatic (100%), while the proportion of asymptomatic concordant cases dropped significantly to 28.1% (p < 0.001).

**Table 4:** Characteristics of concordant and discordant cases at year 1 and year 3

Variable	Year 1 (N = 144)		p	Year 3 (N = 124)		p
	Concordant (n = 87)	Discordant (n = 57)		Concordant (n = 96)	Discordant (n = 28)	
Reflux Symptoms (GerdQ)						
Symptomatic	37(42.5)	0(0)	< *0.001*	69(71.9)	0(0)	< *0.001*
Asymptomatic	50(57.5)	57(100)		27(28.1)	28(100)	
Demography						
Age (preop) M ± SD	35.9 ± 11.4	33.9 ± 11.5	0.308	35.8 ± 11.8	33.3 ± 9.5	0.256
Sex (female)	67(77.0)	42(73.7)	0.798	72(75.0)	22(78.6)	0.798
Smoking	9(10.3)	9(15.8)	0.479	12(12.5)	4(14.3)	0.757
Anthropometry M ± SD						
Weight (kg)	67.3 ± 5.8	67.3 ± 5.3	0.957	67.1 ± 6.3	66.2 ± 4.6	0.393
BMI (kg/m^2^)	25.4 ± 1.9	25.3 ± 1.7	0.815	25.2 ± 1.9	25.2 ± 2.1	0.990
TWL%	42.2 ± 0.3	42.2 ± 0.4	0.520	42.7 ± 0.8	42.6 ± 1.0	0.418
EWL%	99.7 ± 10.3	99.7 ± 8.5	0.963	100.7 ± 10.8	100.5 ± 9.8	0.926
Associated Medical Conditions						
Osteoarthritis	8(9.2)	4(7.0)	0.764	6(6.3)	0(0.0)	0.336
Dyslipidemia	16(18.4)	9(15.8)	0.859	9(9.4)	3(10.7)	0.732
Diabetes	1(1.1)	0(0.0)	1.000	0(0.0)	0(0.0)	1.000
Hypertension	8(9.2)	6(10.5)	0.782	6(6.3)	3(10.7)	0.421
Sleep apnea	2(2.3)	2(3.5)	0.648	1(1.0)	0(0.0)	1.000
Cardiac	3(3.4)	1(1.8)	1.000	1(1.0)	0(0.0)	1.000

Cell values represent frequency (%) unless otherwise stated; *M* ± *SD* mean ± standard deviation; *GerdQ* GERD symptoms frequency questionnaire; *preop* preoperative; *BMI* body mass index; *TWL%* percent total weight loss; *EWL%* percent excess weight loss; Italicized bolded cells indicate statistical significance

Laboratory profiles are provided in Supplementary Table 2. Univariate analyses showed that discordant cases had significantly lower serum glutamic-pyruvic transaminase (SGPT) at year 1 (p = 0.230). At year 3, discordant cases exhibited higher hemoglobin levels (p < 0.001) alongside lower values for free triiodothyronine (FT3) (p = 0.081), thyroid-stimulating hormone (TSH) (p = 0.036), and parathyroid hormone (PTH) (p = 0.147).

### Predictors of Probability of Being Discordant

The GEE analysis presented in Table 5 indicated significant temporal variations in the probability of being discordant post-surgery. Alongside time as a categorical variable (year 1 and year 3), the model incorporated variables selected based on p-values < 0.25 in the univariate analyses, as recommended in the literature [37]. These variables included hemoglobin, ALT, FT3, TSH, and PTH. Findings indicated that the odds of discordance were approximately three times higher at year 1 compared to year 3 (OR = 2.81, 95% CI = 1.64–4.80, p < 0.001). In addition, hemoglobin levels positively correlated with an increased odds ratio of 1.38 for discordance (95% CI = 1.10–1.71, p = 0.004). All other variables were not statistically significant.

**Table 5:** Patient and time-point characteristics as predictors of probability of being discordant

Variable	OR	95% CI	p
Intercept^*a*^	0.45	0.01–26.15	0.703
Year 1 vs year 3	2.81	1.64–4.80	< *0.001*
Hemoglobin	1.38	1.10−1.71	*0.004*
ALT	0.96	0.91–1.02	0.218
FT3	0.77	0.49–1.21	0.263
TSH	0.88	0.65–1.18	0.390
Parathyroid hormone	0.98	0.95–1.02	0.342

Generalized estimating equation analysis;^*a*^Odds of discordance preoperative with all predictors = 0; *OR* odds ratio; *CI* confidence interval; *FT3* free triiodothyronine; *ALT* Alanine Aminotransferase; Italicized cells indicate statistical significance

### Proton Pump Inhibitors (PPI) use

All patients received prophylactic PPI therapy for 3 months postoperatively. By year 1 follow-up, 25.7% of patients were symptomatic (GerdQ ≥ 8) and were maintained on PPI beyond the initial prophylaxis. In addition, 65.3% of patients had abnormal findings on UE and/or biopsy at 1 year; all such patients received combined therapy with a PPI and the mucosal protectant sucralfate. There was substantial overlap between these groups, as essentially all symptomatic patients also exhibited abnormal UE or histopathological findings at 1 year. Overall, roughly 65% of the cohort was on ongoing acid suppression and/or sucralfate therapy at year 1 post-OAGB. By year 3, these proportions had increased: 55.6% of patients reported GERD symptoms (all on PPI) and 78.2% had abnormal UE or biopsy results (all on PPI + sucralfate), corresponding to approximately 78% of the cohort receiving medical therapy, up from ~ 65% at year 1. This indicates a higher utilization of PPI (with or without sucralfate) at postoperative year 3 compared to year 1.

## Discussion

The prevalence of OAGB has surged over the last decade, positioning it as the third most common MBS [38]. Despite the concerns about the high incidence of long-term GERD following bypass procedures, the long gastric pouch and wide anastomosis characteristic of OAGB has been proposed to create a low-pressure environment that minimizes the retrograde flow of gastric contents into the esophagus [39, 40]. Nonetheless, post-OAGB reflux and related abnormalities remain contentious within the surgical community.

The temporal changes and interrelations between post-OAGB reflux symptoms, macroscopic, and microscopic findings have not been systematically evaluated across short- and mid-term intervals. Understanding the dynamics of reflux symptomatology and related macroscopic and microscopic changes over time is critical for building an evidence base that addresses the ongoing debates surrounding post-OAGB reflux and associated complications. This study is the first to systematically address these complex issues, primarily aimed to contribute to bridging that knowledge gap.

We noted a nearly two-fold increase in the proportion of symptomatic patients from year 1 to year 3, with significantly less increase in UE and histopathology abnormalities. This raises the concern of 'clinically silent' patients who, despite being asymptomatic, demonstrated UE and histopathology abnormalities, underscoring the importance of continuous monitoring and in-depth comprehension of the relationships within the GERD triad.

Notably, while symptomatic patients nearly doubled from 25.7% at year 1 to 55.6% at year 3, existing literature from Australia indicated that, in comparison to sleeve gastrectomy and Roux-en-Y gastric bypass, OAGB was the only procedure showing a statistically significant reduction in GerdQ scores six months post-operatively [30]. However, it remains unclear if their cohort included patients with preoperative GERD or symptoms, unlike our series, which exclusively consisted of asymptomatic, GERD-free patients at baseline.

The Lyon Consensus 2.0 reinforces that GERD symptoms alone are insufficient for diagnosis [41], emphasizing the need for objective parameters such as acid exposure time and baseline mucosal impedance. The increasing discordance between symptoms, endoscopic, and histopathological findings in our study aligns with this concept, suggesting that a subset of patients may exhibit pathological reflux despite the absence of overt symptoms.

Pertaining to the relationships between the GERD triad components, we classified the cases into concordant or discordant, which yielded pivotal insights. Discordant cases were consistently asymptomatic at year 1 and year 3, hindering their clinical detection despite abnormalities in one or more diagnostic tests. This discordance highlights the intricate dynamics of the symptoms-UE-histopathology relationship and emphasizes the limitations inherent in relying exclusively on symptomatic evaluations to identify and manage post-OAGB reflux. Previous research has corroborated the weak correlation between symptoms and objective findings, where 17%−28% of endoscopy-confirmed post-OAGB ulcers were asymptomatic [42–44]. Furthermore, an endoscopic and histopathological evaluation conducted two years post-OAGB yielded a 10.3% incidence of marginal ulcers identified via UE, despite that only half the patients presented with symptoms [8]. Pertaining specifically to gastric and esophageal reflux, research integrating endoscopic, biochemical, and histological analyses of 28 patients undergoing OAGB or RYGB found a poor correlation between patient-reported symptoms and objective findings [45]. In addition, some bariatric patients might have preoperative asymptomatic/silent GERD which adds further challenge to the assessment of post-OAGB GERD and raises the attention to the importance of pre-op UE [46–48].

These findings underscore a pressing need for bariatric teams to proactively inquire about reflux after OAGB, as symptoms should not solely direct the assessments for reflux. Our findings also suggest that traditional UE assessment alone may be inadequate in identifying true reflux pathology, reinforcing the need for histopathological analysis and the utilization of more histological markers in line with the Lyon Consensus 2.0 in post-OAGB GERD surveillance [41].

No published studies conducted a simultaneous and thorough analysis of the symptoms-UE-histopathology triad across different time points following OAGB using a sizable cohort. In Brazil, a limited cohort of 39 patients presented some 'relationships' concerning UE findings before surgery and two years after surgery, focusing exclusively on UE findings without comparable histopathological or symptom data [8]. Similarly, in Finland, an investigation into post-OAGB bile reflux among 40 patients assessed histological findings preoperatively and at only one post-operative milestone (six months), again lacking parallel evaluations of UE/macroscopic findings or symptoms throughout these time points [49]. While such published assessments provide valuable insights, they carry inherent limitations as the examination of a single postoperative point offers only a single snapshot of patient status, obscuring subsequent changes and leaving a potential 'blind spot'. In addition, the practice of matching only two components of the triad risks creating knowledge gaps. Such isolated approaches to analyzing the components of the symptoms-UE-histopathology triad deliver a limited perspective on the continuous processes and pathways underlying this complex interplay and fail to capture the interrelated nature of these components as they evolve in concert post-operatively. A more integrated analysis is essential for a comprehensive understanding of these dynamics to decipher and contextualize these relationships effectively.

In addressing the third objective related to patient subsets, it is evident that concordant patients, whether asymptomatic or symptomatic were generally less challenging to identify. Asymptomatic concordant patients present no immediate risk, as indicated by their negative UE and histopathology results; conversely, concordant symptomatic patients, present overt clinical signs that alert practitioners to potential underlying issues, facilitating timely intervention.

In contrast, discordant patients appear to be more potentially ‘deceptive’, particularly the asymptomatic discordant patients, as their symptoms do not align with their UE/histopathology findings, posing a high risk of being overlooked by practitioners not maintaining a high index of suspicion.

Regarding the fourth objective of patient characteristics, it remains impractical to accurately predict which post-OAGB patients are more likely to experience discordance. Current demographic and anthropometric metrics, including weight loss, BMI, TWL%, and EWL%, along with associated medical conditions and various laboratory biomarkers did not indicate the likelihood of discordance at either year 1 or year 3 after surgery. Our findings resonate with existing literature highlighting that the underlying reasons for variability in symptom severity and complications related to reflux among individuals are inadequately understood [50–52]. Furthermore, they corroborate the challenges involved in identifying the ‘at-risk’ patients for post-OAGB reflux, underscoring the necessity for comprehensive detection strategies that expand beyond traditional clinical markers. To delineate the traits of post-OAGB patients predisposed to discordance, sustained longitudinal follow-up is essential. This aligns with the consensus that the long-term GERD after MBS poses significant diagnostic complexities [39].

On the question of the critical time points warranting heightened vigilance for post-OAGB reflux-related abnormalities, we noted that the year 1 follow-up mark demonstrated a three-fold increased likelihood of discordance within the symptoms-UE-histopathology triad when compared to the year 3 follow-up. Thus, the year 1 milestone represents a crucial follow-up time mark and a pivotal opportunity for clinicians to closely monitor post-OAGB patients to detect reflux and related abnormalities and actively seek out discordant patients. Notably, elevated hemoglobin levels correlated with an increased 1.38 odds of discordance. However, there is no available clinical evidence that support or oppose this finding.

Previous studies have documented the weak correlation between clinical symptoms and objective findings in post-OAGB reflux cases. For example, bile reflux often precipitates histological gastritis but does not consistently equate to adverse symptomatic presentations [53]. Similarly, in other bariatric procedures, asymptomatic BE was found in about 21% of post-sleeve gastrectomy patients [54]. While this recognition of poor correlation is noteworthy, it lacks actionable tools for practitioners. More nuanced prompts and evaluative cues are needed. The current study's exploration of the concept of discordance and concordance within the GERD triad is a novel notion in advancing our understanding of the patient experience post-OAGB by elucidating the complex interrelationships among these factors. With this enhanced awareness, bariatric teams may better monitor patients to prevent those at risk from being overlooked.

This study has limitations. It was conducted at a single center. In addition, subclassification based purely on symptoms may have led to underdiagnosis of clinically significant reflux disease, given the high discordance rate observed in our study. Implementing Lyon Consensus 2.0 criteria could help refine post-OAGB patient classification, ensuring that those with histological or endoscopic evidence of reflux-related injury receive appropriate long-term surveillance and intervention [41]. It would have been useful to use objective tools such as scintigraphy, pH-impedance, baseline mucosal impedance and manometry, which could enhance the assessment of reflux, particularly in asymptomatic or discordant patients, and differentiate between acid and biliary reflux. The Lyon Consensus 2.0 emphasizes that GERD classification should integrate objective parameters such as pH-impedance monitoring and histopathological markers [41]. Another limitation was that the post-operative use of acid suppression at the time of data collection was not held before UE. This could be responsible for the high rate of discordance between symptoms and endoscopic or histopathological findings. Whilst some authors advocate stopping PPI and H2 blockers two weeks before completing the questionnaire, and also before conducting UE in patients who were using these medications [19] to prevent masking of findings, however, two weeks might appear not to be sufficient for pathology to revert/develop. An extended follow-up period with further endoscopic routine surveillance would have been useful in providing more insights into the changes in the GERD triad over time. Given the dramatic change in the incidence of asymptomatic and discordant patients between year 1 and year 3 in this study, assessments beyond year 3 might reveal further changes. The ASMBS recommends that clinicians should consider a screening UE at 3 or more years even with absence of symptoms after certain bariatric procedures, and further EGD screening every 5 years thereafter [29].

Despite these limitations, the current study has remarkable strengths, particularly as it represents a pioneering effort to investigate the longitudinal outcomes of patients after OAGB in terms of subjective reflux symptoms and objective diagnostic abnormalities. The study analyzed the dynamic interplay within the GERD triad, across a substantial cohort at both short-term and mid-term intervals after OAGB. The characterization of patients through the lens of the GERD triad introduced a novel conceptual framework distinguishing between concordant and discordant cases, laying the groundwork for deeper insights into the complexities of post-OAGB reflux phenomena. Furthermore, the study analysed whether the distinguishing features of concordant versus discordant patients varied across a comprehensive array of variables, to identify at-risk patients early. The analysis also assessed potential correlations between specific patient characteristics and time points, pertaining to the likelihood of experiencing discordance.

## Future Considerations

While our findings provide critical insights, several aspects warrant further exploration. Future research should integrate Lyon Consensus 2.0 diagnostic thresholds to distinguish between physiological and pathological post-OAGB reflux [41], refining the postoperative surveillance strategies, and improving predictive models for long-term GERD-related complications.

In addition, longer-term studies are needed to assess whether histopathological changes progress, stabilize, or regress beyond three years, identifying potential risk factors for persistent or worsening reflux disease. Moreover, the impact of surgical variations, including limb length modifications should also be evaluated to determine their role in reflux severity and progression [55].

Given the high prevalence of discordant cases, future efforts should focus on developing personalized risk stratification models that integrate symptom profiles, endoscopic changes, histopathological changes, and objective reflux diagnostic tools. This approach would ultimately guide individualized precision treatment plans for patients with persistent reflux despite normal symptomatology, ensuring optimized long-term management.

## Conclusion

The evolution of the symptoms-UE-histopathology triad post-OAGB demonstrates significant variability. The year 1 data revealed a significant one-third lower probability of symptom development compared to the year 3 findings; however, there was a threefold increase in the likelihood of discordance within the symptoms-UE-histopathology triad. This underscores the necessity for more vigilant monitoring of post-OAGB patients and extending the follow-up of such patients beyond three years.

## Supplementary Information

Below is the link to the electronic supplementary material.

Supplementary file1 (DOCX 34.9 KB)

## Data Availability

Data can be available upon reasonable request and agreement of the institution where the research was undertaken.
