# Supplementary material for: Concordant and Discordant Interrelationships of the GERD Triad of Symptoms, Endoscopy Findings, and Histopathological Changes Over Time after One Anastomosis Gastric Bypass
Source: Obes Surg. 2025 Nov 18;35(12):5080–91. doi: 10.1007/s11695-025-08277-7 (PMC12722256; doi:10.1007/s11695-025-08277-7)
Supplement: Supplementary file 1 — Supplementary file1 (DOCX 34.9 KB) [file 11695_2025_8277_MOESM1_ESM.docx]

**Supplementary Box 1.** OAGB Surgical Technique

| All surgical procedures were undertaken by one experienced bariatric team. The patient was positioned in modified lithotomy, with five trocars introduced through standard ports, with the surgeon standing between the patient’s legs. Initial dissection of the lesser omentum below the crow's foot facilitated the insertion of the stapler. A gastric pouch of a minimum length of 15 cm was created above the gastrojejunostomy using a linear stapler and reloads, over a 40-Fr bougie. The first reload was deployed transversely beneath the incisura angularis, followed by vertical reloads towards the angle of His, requiring dissection of this anatomical landmark. For the biliopancreatic limb, lengths ranged from 150-200 cm (200 cm utilized for patients with a BMI exceeding 50 kg/m²), ensuring a common limb length of at least 300 cm. Blue reloads were employed for constructing the gastrojejunostomy, with stapling defects secured using continuous sutures. Crural repair for diagnosed hiatal hernia or concomitant cholecystectomy was attempted in all relevantly diagnosed and indicated cases. An intraoperative methylene blue leak test was routinely conducted to ensure the integrity of the anastomosis |
| --- |

**Supplementary Table 1.** Endoscopy and biopsy findings of the sample at years 1 and 3

| **Finding** | **Year 1**  (n=144) | **Year 3**  (n=124) |
| --- | --- | --- |
| **Endoscopy** |  |  |
| **Distal Esophagus** |  |  |
| Normal | 116(75.7) | 115(92.7) |
| Abnormal | 28(24.3) | 9(7.3) |
| Hiatus Hernia | 3(2.1) | 4(3.2) |
| Incompetent Cardia | 24(16.7) | 4(3.2) |
| Mucosal Abnormality | 4(2.8) | 4(3.2) |
| Bile | 6(4.2) | 5(4.0) |
| **Gastric pouch** |  |  |
| Normal | 61(42.4) | 33(26.6) |
| Abnormal | 83(57.6) | 91(73.4) |
| Non-erosive gastritis | 59(41.0) | 60(48.4) |
| Erosive gastritis | 24(16.7) | 31(25.0) |
| **Anastomotic site** |  |  |
| Normal | 121(84.0) | 97(78.2) |
| Abnormal | 23(16.0) | 27(21.7) |
| Ulcer | 3(2.1) | 4(3.2) |
| Mucosal hyperemia | 20(13.9) | 23(18.5) |
| **Biopsy** |  |  |
| **Distal esophagus** |  |  |
| Normal | 53(36.8) | 34(27.4) |
| Abnormal | 91(63.2) | 90(72.6) |
| Chronic Inflammation | 91(63.2) | 90(72.6) |
| Barrett’s esophagus | 0(0) | 1(0.8) |
| **Gastric Pouch** |  |  |
| Normal | 77(53.5) | 31(21.5) |
| Abnormal | 67(46.5) | 93(75) |
| Chronic Gastritis | 66(45.8) | 91(73.4) |
| Active (erosive) | 3(2.1) | 4(3.2) |
| Non-active | 66(45.8) | 91(73.4) |
| H. Pylori | 6(4.2) | 8(6.5) |
| **Anastomotic Site** |  |  |
| Normal | 121(84.0) | 97(78.2) |
| Abnormal | 23(16) | 27(21.7) |
| Chronic Inflammation | 20(13.9) | 23(18.5) |
| Ulcer | 3(2.1) | 4(3.2) |

Cell values represent frequency (%)

**Supplementary Table 2.** Laboratory profile of concordant and discordant cases at years 1 and 3

| **Variable** | **Year 1** (N=144) | | **p** | **Year 3** (N=124) | | **p** |
| --- | --- | --- | --- | --- | --- | --- |
|  | **Concordant**  (n=87) | **Discordant**  (n=57) |  | **Concordant**  (n=96) | **Discordant**  (n=28) |  |
| **Laboratory investigations** | | | | | | |
| Hemoglobin | 12.4±1.2 | 12.4±1.0 | 0.751 | 12.5±1.4 | 13.7±0.7 | ***< 0.001*** |
| Ferritin | 121.9±9.0 | 122.9±7.9 | 0.451 | 139.8±24.9 | 134.9±23.5 | 0.341 |
| WBC | 5.1±0.9 | 5.1±0.8 | 0.790 | 4.9±0.8 | 5.0±0.6 | 0.622 |
| SGOT | 26.5±4.7 | 25.9±4.3 | 0.366 | 26.3±4.5 | 26.8±4.1 | 0.594 |
| SGPT | 40.2±4.8 | 39.2±4.5 | ***0.230*** | 39.5±4.6 | 39.7±4.7 | 0.874 |
| Urea | 33.5±8.1 | 33.0±7.9 | 0.710 | 33.4±7.6 | 33.5±8.0 | 0.988 |
| Creatinine | 1.1±0.3 | 1.1±0.3 | 0.894 | 0.9±0.1 | 0.9±0.2 | 0.342 |
| FT3 | 4.4±0.6 | 4.4±0.7 | 0.788 | 4.3±0.6 | 4.1±0.6 | *0.081* |
| FT4 | 20.6±3.9 | 20.5±4.3 | 0.937 | 20.7±3.9 | 21.0±4.2 | 0.762 |
| TSH | 2.3±0.9 | 2.4±0.9 | 0.697 | 2.4±0.9 | 2.1±0.7 | ***0.036*** |
| Fasting glucose | 81.4±7.4 | 81.6±7.3 | 0.858 | 74.7±6.6 | 75.8±6.8 | 0.466 |
| HbA1c | 4.7±0.8 | 4.9±0.7 | 0.315 | 4.5±0.5 | 4.5±0.6 | 0.931 |
| Cholesterol | 155.7±32.6 | 151.9±34.0 | 0.507 | 116.7±31.6 | 128.3±30.1 | 0.081 |
| Triglycerides | 112.5±19.6 | 112.4±17.7 | 0.971 | 92.1±16.5 | 91.4±17.5 | 0.840 |
| LDL | 70.4±18.8 | 68.6±17.8 | 0.561 | 60.4±14.5 | 58.5±14.6 | 0.545 |
| Albumin | 4.2±0.4 | 4.2±0.3 | 0.755 | 4.3±0.4 | 4.4±0.4 | 0.360 |
| Calcium | 9.4±0.4 | 9.4±0.5 | 0.504 | 9.5±0.4 | 9.6±0.6 | 0.676 |
| Vitamin D | 34.9±5.8 | 34.3±5.7 | 0.597 | 39.9±6.4 | 39.5±4.7 | 0.727 |
| Vitamin B12 | 621.3±145.8 | 636.4±181.4 | 0.601 | 699.6±142.4 | 720.6±139.8 | 0.490 |
| Parathormone | 40.3±7.7 | 40.5±7.8 | 0.854 | 41.0±7.1 | 39.1±5.4 | ***0.147*** |

Cell values represent mean±standard deviation; *WBC* white blood cells; *SGOT* serum glutamic-oxaloacetic transaminase (aspartate transaminase); *SGPT* serum glutamic pyruvic transaminase; *FT3* free triiodothyronine; *FT4* free thyroxine; *LDL* low density lipoproteins; *TSH* thyroid stimulating hormone; Italicized bolded cells indicate statistical significance (p <0.25 in univariate analyses), as covariates with weak univariate associations may significantly impact the model when combined [37]
